# Relationship between basal sodium intake and the effects of dapagliflozin in albuminuric diabetic kidney disease

**DOI:** 10.1038/s41598-020-79687-z

**Published:** 2021-01-13

**Authors:** Sho Kinguchi, Hiromichi Wakui, Yuzuru Ito, Yoshinobu Kondo, Kengo Azushima, Uru Osada, Tadashi Yamakawa, Tamio Iwamoto, Jun Yutoh, Toshihiro Misumi, Gen Yasuda, Taishi Yoshii, Kotaro Haruhara, Yusuke Kobayashi, Takeharu Yamanaka, Yasuo Terauchi, Kouichi Tamura

**Affiliations:** 1 Department of Medical Science and Cardiorenal Medicine, Yokohama City University Graduate School of Medicine, 3-9 Fukuura, Kanazawa-ku, Yokohama, 236-0004 Japan; 2 Department of Endocrinology and Metabolism, Yokohama City University Graduate School of Medicine, 3-9 Fukuura, Kanazawa-ku, Yokohama, 236-0004 Japan; 3 Department of Diabetes and Endocrinology, Saiseikai Yokohama South Hospital, Yokohama, Japan; 4 Department of Endocrinology and Diabetes, Yokohama City University Center Hospital, Yokohama, Japan; 5 Department of Nephrology and Hypertension, Saiseikai Yokohama South Hospital, Yokohama, Japan; 6 Department of Nephrology and Hypertension, Yokohama Minami Kyousai Hospital, Yokohama, Japan; 7 Department of Biostatistics and Epidemiology, Yokohama City University Graduate School of Medicine, Yokohama, Japan; 8 Department of Nephrology and Hypertension, Yokohama City University Center Hospital, Yokohama, Japan; 9 Department of Endocrinology and Metabolism, Yokohama Minami Kyousai Hospital, Yokohama, Japan; 10 Center for Novel and Exploratory Clinical Trials (Y-NEXT), Yokohama City University, Yokohama, Japan

**Keywords:** Chronic kidney disease, Endocrinology

## Abstract

We investigated the impact of basal dietary sodium intake on the dapagliflozin-induced changes in albuminuria and blood pressure (BP) measured at home in patients with diabetic kidney disease (DKD).This was a secondary analysis of the Y-AIDA Study, in which DKD patients with estimated glomerular filtration rate (eGFR) ≥ 45 ml/min/1.73 m^2^ and urinary albumin-to-creatinine ratio (UACR) ≥ 30 mg/g creatinine were administered dapagliflozin for 24 weeks, and dapagliflozin significantly improved albuminuria levels and home BP profiles. The effects on UACR, home-measured BP, and eGFR were compared between high- and low-sodium intake groups (HS and LS groups), which were created using baseline urinary sodium-to-creatinine ratio of 84 participants with available basal sodium-to-creatinine ratios. At baseline, clinic-/home-measured BPs, UACR, and eGFR, were comparable in the two groups. After 24 weeks, the reductions from baseline in ln-UACR were comparable in the two groups. In contrast, the reductions in evening home systolic BP and eGFR from baseline were larger in HS than in LS (BP: − 13 ± 2.08 vs. − 6 ± 1.88, *P* = 0.020; eGFR: − 3.33 ± 1.32 vs. 0.37 ± 1.29, *P* = 0.049). The home BP-lowering effects of dapagliflozin are larger in HS than LS, concomitant with a larger reduction in eGFR, suggesting a dapagliflozin-induced improvement in glomerular relative hyperfiltration in HS.

## Introduction

Diabetic kidney disease (DKD) is a common comorbidity in patients with type 2 diabetes mellitus (T2DM), which is the leading cause of end-stage renal disease (ESRD)^1,2^. DKD is conventionally thought to predominantly affect the glomerulus, through intraglomerular hypertension, which promotes glomerulosclerosis and inflammation especially in the context of systemic hypertension^3,4^. From a renal mechanistic perspective, sodium plays an important role in the pathogenesis of DKD. High dietary sodium intake and volume expansion are risk factors for hypertension, which promotes the progression of DKD^3,5^. Furthermore, aberrant proximal tubular handling of sodium plays an important role in the pathogenesis of glomerular hyperfiltration^6^, which contributes to the increase in urinary albumin excretion and the progressive decline in renal function^7^.

High sodium intake is associated with high blood pressure (BP), a risk factor for cardiovascular and renal diseases^8^, and the restriction of sodium intake lowers BP^9–11^. High sodium intake also causes resistant hypertension^12^. Some previous studies have also demonstrated that many Japanese patients with DKD have salt-sensitive hypertension^13^. Furthermore, high dietary sodium intake is associated with a high incidence of cardiovascular disease in T2DM patients^14^. The renal and cardiovascular protective effects of conventional therapy, which includes an angiotensin receptor blocker (ARB), were superior to those of non-renin-angiotensin system (RAS) inhibitor-based therapy in T2DM patients with high sodium intake^15^.

Dapagliflozin is a sodium-glucose cotransporter-2 (SGLT2) inhibitor that increases urinary glucose excretion and reduces hemoglobin A1c (HbA1c)^16^. SGLT2 inhibitors are also reported to have pleiotropic effects, including reductions in body mass, BP, and urinary albumin excretion, which have cardio-renal protective effects^16–20^. The inhibition of SGLT2 also increases urinary sodium excretion, and this natriuretic effect of SGLT2 inhibitors may be clinically important^3,21,22^. Recently, we reported that dapagliflozin improves albuminuria and the BP profile assessed at home, and the latter is associated with the amelioration of albuminuria in Japanese DKD patients^23^. Therefore, we hypothesized that the improvements in albuminuria and home BP profile that are associated with dapagliflozin may be more pronounced in Japanese DKD patients with high sodium intake.

The purpose of the present study was to investigate the impacts of basal dietary sodium intake, estimated using urinary sodium excretion, on the improvements in urinary albumin-to-creatinine ratio (UACR) and home BP associated with the use of dapagliflozin in Japanese DKD patients. To this end, data from the Yokohama Add-on Inhibitory efficacy of Dapagliflozin on Albuminuria in Japanese patients with type 2 diabetes study (Y-AIDA study)^23^ were analyzed.

## Methods

### Study design

We conducted a secondary analysis of data from the Y-AIDA study^23^, which was a prospective, multi-center, single-arm study that was performed as previously described^23^. Briefly, 86 T2DM patients with HbA1c 7.0–10.0%, estimated glomerular filtration rate (eGFR) ≥ 45 ml/min/1.73 m^2^, and urine albumin‐to‐creatinine ratio (UACR) ≥ 30 mg/g creatinine (gCr) were enrolled, and 85 of these patients were administered add-on dapagliflozin for 24 weeks^23^. This secondary analysis included 84 participants whose basal urinary sodium-to-creatinine ratios were available. The study complied with the ethical principles of the Declaration of Helsinki. Ethical approval was obtained from the ethical review boards of Yokohama City University (B150305009), Yokohama City University Center Hospital, Saiseikai Yokohama South Hospital and Yokohama Minami Kyousai Hospital. The study is registered in the UMIN Clinical Trials Registry (UMIN000018930; http://www.umin.ac.jp/ctr/index-j.htm). All the participants provided written informed consent prior to their participation.

### Physical findings and laboratory measurements

Body mass, body mass index (BMI), and BP were measured, and blood and urine sampling was performed in the clinic in the fasted state, at baseline (0 weeks) and after 8 weeks, 16 weeks, and 24 weeks of treatment, as described previously^23^. Urinalysis was performed in spot urine samples at the applicable visits. At baseline, urine sodium (mEq/L) was measured using the electrode method, and urine creatinine (mg/dL) was measured using an enzymatic method, by SRL Inc. (Tokyo, Japan).

### Home BP and nocturnal home BP measurements

Home BP and nocturnal home BP were measured using a validated and internet-interfaced BP monitor (HEM-7252G-HP, Omron Corporation, Kyoto), as previously described^23^. The home BP monitoring device enabled automatic transmission of all home BP readings to a server after each measurement, using a mobile telecommunication system (MedicalLINK), to allow accurate analysis of the home BP profile^24,25^. Three home BP readings were taken at 1-min intervals in a sitting position in the morning and evening during the 7-day study periods before the 0-week and 24-week visits. Morning BP was measured before breakfast, within 1 h of waking, and before taking anti-hypertensive medication. Evening BP was measured before going to bed. The morning and evening home BPs were defined as the mean BP value during the mornings and evenings of the 7-day study periods, which were the means of the three readings obtained in the morning and evening of each day. The HEM-7252G-HP monitor can also record BP readings at fixed times, and was pre-set to record nocturnal home BP measurements at 02:00, 03:00, 04:00, and 05:00 h. Nocturnal home BPs were also measured during the 7-day study period before the 0- and 24-week visits. Nocturnal home BP was defined as the mean nighttime BP during the 7-day study periods, which represented the mean of four readings per night.

### Statistical analysis

To assess the effects of baseline dietary sodium intake on clinical variables during add-on dapagliflozin administration, participants were allocated to two groups according to their median urinary sodium-to-creatinine ratio at baseline. The median urinary sodium-to-creatinine ratio at baseline was 1.14 (× 10^2^ mEq/g Cr) (interquartile range (IQR): 0.815, 1.595). Therefore, we allocated participants with urinary sodium-to-creatinine ratios > 1.14 to the high-sodium intake group (HS group) and the other participants, who had urinary sodium-to-creatinine ratios < 1.14, to the low-sodium intake group (LS group). Their estimated daily salt intakes (g/day) were also calculated using the Tanaka formula^26^. The Tanaka formula is as follows: 24-h urinary sodium (Na) excretion (mEq/day) = 21.98 × {[Spot urinary sodium concentration (mEq/L) ÷ (Spot urinary creatinine concentration (mg/dL) × 10)] × predicted 24-h urinary creatinine excretion (PrUCr24h) (mg/day)}^0.392^; PrUCr24h (mg/day) = 14.89 × weight (kg) + 16.14 × height (cm) − 2.04 × age (years) – 2,244.45. Estimated daily salt intake (g/day) = 24-h urinary sodium excretion (mEq/day) ÷ 17.

Furthermore, participants were allocated to two groups according to median estimated daily salt intake (g/day) calculated using the Tanaka formula at baseline. Median estimated daily salt intake at baseline was 9.213 (g/day) (IQR: 8.063, 10.446). Therefore, we allocated participants with estimated daily salt intake > 9.213 to the high salt intake group, and those with estimated daily salt intake < 9.213 to the low salt intake group. Changes in natural logarithm of UACR (ln-UACR), eGFR, and home BP from baseline were compared between the high salt intake and low salt intake groups.

The data in the text, figures, and tables are presented as mean ± standard error (SE), percentage, or median (IQR). For statistical analyses of the differences between the high-sodium intake and low-sodium intake groups, unpaired *t*-tests was performed for continuous variables and Fisher’s exact test was performed for categorical variables, at baseline and at each subsequent visit. Differences in UACR at baseline were analyzed using the Mann–Whitney *U* test. Statistical analysis was performed using SAS version 9.4 (SAS Institute Japan) at the Department of Biostatistics and Epidemiology in Yokohama City University Graduate School of Medicine, and a value of *P* < 0.05 was considered to represent statistical significance.

## Results

### Baseline patient characteristics of the HS and LS groups

Eighty-five participants were administered add-on dapagliflozin for 24 weeks in the Y-AIDA study, as described previously^23^. The participants were divided into two groups (the HS and LS groups) according to their median basal urinary sodium-to-creatinine ratio (Table 1, 2). The duration of diabetes was longer (14.8 ± 1.6 vs. 10.3 ± 1.2 years, *P* = 0.027) and HbA1c was higher (8.0 ± 0.1 vs. 7.7 ± 0.1, *P* = 0.029) in the HS group than in the LS group. The other parameters (body mass, BMI, UACR, and eGFR) were comparable in the two groups (Table 1). Median UACR was 199.5 mg/g Cr (IQR: 61.4, 629.0) in the HS group, and 102.5 mg/g Cr (IQR: 40.1, 666.0) in the LS group (*P* = 0.310). There were no significant differences in the concomitant use of antidiabetic agents or antihypertensive agents, including RAS inhibitors and diuretics, between the two groups (Table 2).

**Table 1:** Baseline characteristics of the participants.

Variable	HS group N = 42	LS group N = 42	*P* value
Urinary Na/Cr ratio (× 10^2^ mEq/g Cr)	1.85 ± 0.11	0.76 ± 0.05	< 0.001
Estimated daily salt intake (g/day)	10.88 ± 0.29	7.80 ± 0.27	< 0.001
Age (years)	65.6 ± 1.5	64.9 ± 1.6	0.772
Sex (male/female)	28/14	36/6	0.071
Body mass index (kg/m^2^)	27.5 ± 0.8	26.8 ± 0.6	0.512
Duration of diabetes (years)	14.8 ± 1.6	10.3 ± 1.2	0.027
Hypertension n (%)	36 (85.7)	37 (92.5)	0.483
Dyslipidemia n (%)	36 (85.7)	35 (87.5)	1.000
Hyperuricemia n (%)	6 (14.3)	13 (32.5)	0.068
Angina pectoris n (%)	5 (12.2)	4 (9.5)	0.738
Myocardial infarction n (%)	2 (4.9)	0 (0.0)	0.241
Stroke n (%)	4 (9.8)	7 (16.7)	0.520
**Clinic blood pressure**			
SBP (mmHg)	144 ± 3.3	140 ± 2.9	0.404
DBP (mmHg)	79 ± 2.0	78 ± 1.9	0.845
**Glucose metabolism**			
Fasting blood glucose (mg/dl)	156.7 ± 6.1	158.9 ± 5.6	0.792
HbA1c (%)	8.0 ± 0.1	7.7 ± 0.1	0.029
**Renal function**			
eGFR (ml/min/1.73 m^2^)	69.8 ± 2.7	65.0 ± 2.7	0.219
Median UACR (mg/g Cr) (interquartile range)	199.5 (61.4, 629.0)	102.5 (40.1, 666.0)	0.310

Values are mean ± standard error (SE). UACR is expressed as median (interquartile range). Analyses were performed using unpaired *t*-tests for continuous data and Fisher’s exact tests for categorical data. Mann–Whitney *U* test was used for analysis of UACR.

*HS group* high sodium intake group, *LS group* low sodium intake group, *Na* sodium, *Cr* creatinine, *SBP* systolic blood pressure, *DBP* diastolic blood pressure, *HbA1c* glycated hemoglobin, *eGFR* estimated glomerular filtration rate, *UACR* urine albumin-to-creatinine ratio.

**Table 2:** Baseline medications of the participants.

Variable	HS group N = 42	LS group N = 42	*P* value
**Antidiabetic agents**			
Insulin n (%)	14 (36.8)	13 (37.1)	1.000
Biguanides n (%)	28 (73.7)	20 (57.1)	0.149
DPP-4 inhibitors n (%)	22 (57.9)	24 (68.6)	0.467
Sulfonylureas n (%)	15 (39.5)	8 (22.9)	0.141
α-glucosidase inhibitors n (%)	6 (15.8)	11 (31.4)	0.166
Thiazolidinediones n (%)	4 (10.5)	8 (22.9)	0.211
Glinides n (%)	3 (7.9)	4 (11.4)	0.704
GLP1 agonists n (%)	3 (7.9)	5 (14.3)	0.468
**Antihypertensive agents**			
RAS inhibitors			
Angiotensin II receptor blockers n (%)	28 (66.7)	29 (69.0)	1.000
Angiotensin-converting enzyme inhibitors n (%)	3 (7.1)	1 (2.4)	0.616
Calcium-channel blockers n (%)	27 (64.3)	25 (59.5)	0.823
Diuretics n (%)	6 (14.3)	4 (9.5)	0.738
α1-blockers n (%)	1 (2.4)	1 (2.4)	1.000
β-blockers n (%)	4 (9.5)	3 (7.1)	1.000
Aldosterone antagonist n (%)	1 (2.4)	2 (4.8)	1.000
α2-agonists n (%)	0 (0.0)	2 (4.8)	0.494

Analyses were performed using unpaired *t*-tests for continuous data and Fisher’s exact tests for categorical data.

*HS group* high sodium intake group, *LS group* low sodium intake group, *DPP-4* dipeptidyl peptidase 4, *GLP1* glucagon-like peptide 1, *RAS* renin–angiotensin system.

### Effects of add-on dapagliflozin therapy on renal endpoints in the HS and LS groups

**Figure 1:**
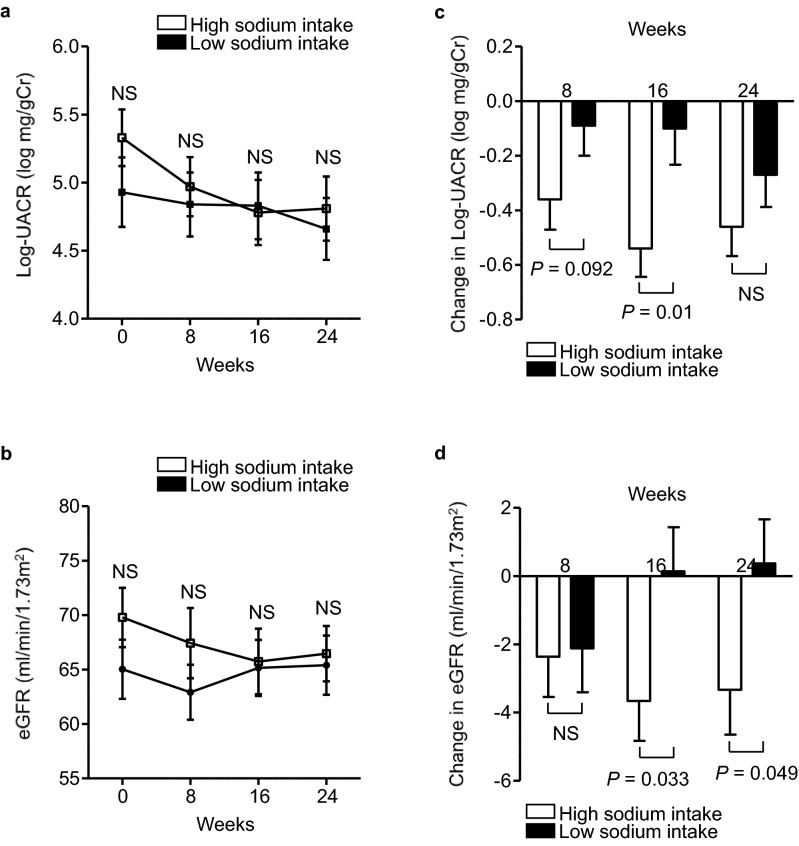
Effects of add-on dapagliflozin therapy on renal endpoints in the HS and LS groups.* (a)* Natural logarithm of urine albumin-to-creatinine ratio (ln-UACR), and **(b)** estimated glomerular filtration (eGFR) in dapagliflozin-treated patients during the 24-week study period. Change in **(c)** ln-UACR, and **(d)** eGFR during the 24-week study period. Eighty-four DKD patients were allocated to a high sodium intake (HS) group (open square) or a low sodium intake (LS) group (filled square), according to their median urinary sodium-to-creatinine ratio at baseline. Values are mean ± SE. The differences in the effects of dapagliflozin treatment between the two groups were analyzed using unpaired *t*-tests at each visit. *NS* not significant.

The natural logarithm of UACR (ln-UACR) and eGFR were similar at each visit in the two groups (Fig. 1a,b). However, regarding the changes from baseline, the reduction in ln-UACR from baseline tended to be larger at week 8 (− 0.36 ± 0.11 vs. − 0.09 ± 0.11, *P* = 0.092), and was significantly larger at week 16 (− 0.54 ± 0.10 vs. − 0.10 ± 0.13, *P* = 0.010) in the HS group. However, after 24 weeks, the reductions from baseline in ln-UACR were comparable in the two groups (− 0.46 ± 0.11 vs. − 0.27 ± 0.12, *P* = 0.237; Fig. 1c). The reduction in eGFR from baseline was similar at week 8 (− 2.36 ± 1.18 vs. − 2.12 ± 1.29, *P* = 0.890), whereas the reductions in eGFR at week 16 and week 24 from baseline were larger in the HS group (week 16: − 3.66 ± 1.17 vs. 0.14 ± 1.29, *P* = 0.033; week 24: − 3.33 ± 1.32 vs. 0.37 ± 1.29, *P* = 0.049; Fig. 1d).

### Effects of add-on dapagliflozin therapy on the glucose metabolism, clinic-measured blood pressure, body mass, and body mass index of the HS and LS groups

**Figure 2:**
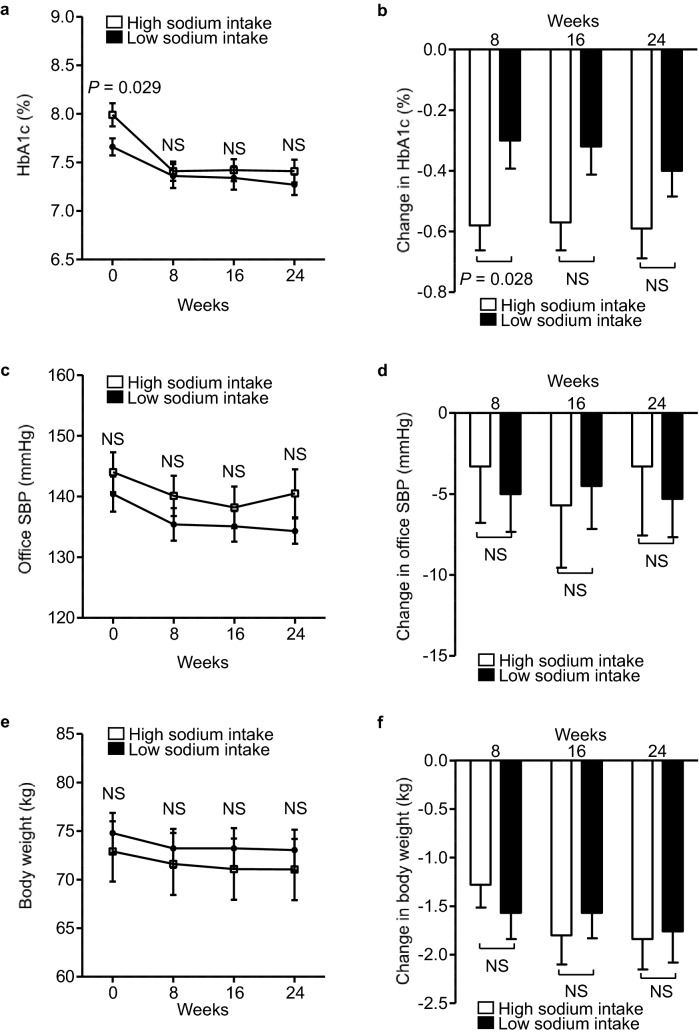
Effects of add-on dapagliflozin therapy on the glucose metabolism, clinic-measured blood pressure, body mass, and body mass index of the HS and LS groups.* (a)* HbA1c, **(c)** fasting blood glucose (FBG), **(e)** clinic systolic blood pressure (SBP), **(g)** clinic diastolic blood pressure (DBP), **(i)** body mass, and **(k) **body mass index (BMI) in dapagliflozin-treated patients during the 24-week study period. Change in **(b)** HbA1c, **(d)** FBG, **(f**) clinic SBP, **(h) **clinic DBP, **(j)** body mass, and **(l)** BMI during the 24-week study period. Eighty-four DKD patients were allocated to a high sodium intake (HS) group (open square) or a low sodium intake (LS) group (filled square), according to their median urinary sodium-to-creatinine ratio at baseline. Values are mean ± SE. The differences in the effects of dapagliflozin treatment between the two groups were analyzed using unpaired *t*-tests at each visit. NS, not significant.

Although HbA1c was significantly higher at baseline in the HS group than in the LS group (7.99 ± 0.12 vs. 7.66 ± 0.09, *P* = 0.029; Fig. 2a), there were no significant differences in HbA1c at weeks 8, 16, and 24 between the two groups. In addition, the reduction in HbA1c from baseline was significantly larger at week 8 in the HS group (− 0.58 ± 0.08 vs. − 0.30 ± 0.09, *P* = 0.028; Fig. 2b). At weeks 16 and 24, the reductions in HbA1c from baseline were similar in the two groups (week 16: − 0.57 ± 0.09 vs. − 0.32 ± 0.09, *P* = 0.057; week 24: − 0.59 ± 0.10 vs. − 0.40 ± 0.08, *P* = 0.148; Fig. 2b). However, there were no significant differences in fasting blood glucose (FBG), clinic systolic BP, clinic diastolic BP, body mass, and BMI between the two groups at each visit (Fig. 2c,e). Similarly, the reductions in these parameters from baseline were comparable at each visit in the two groups (Fig. 2d,f).

### Effects of add-on dapagliflozin therapy on home-measured blood pressure in the HS and LS groups

**Figure 3:**
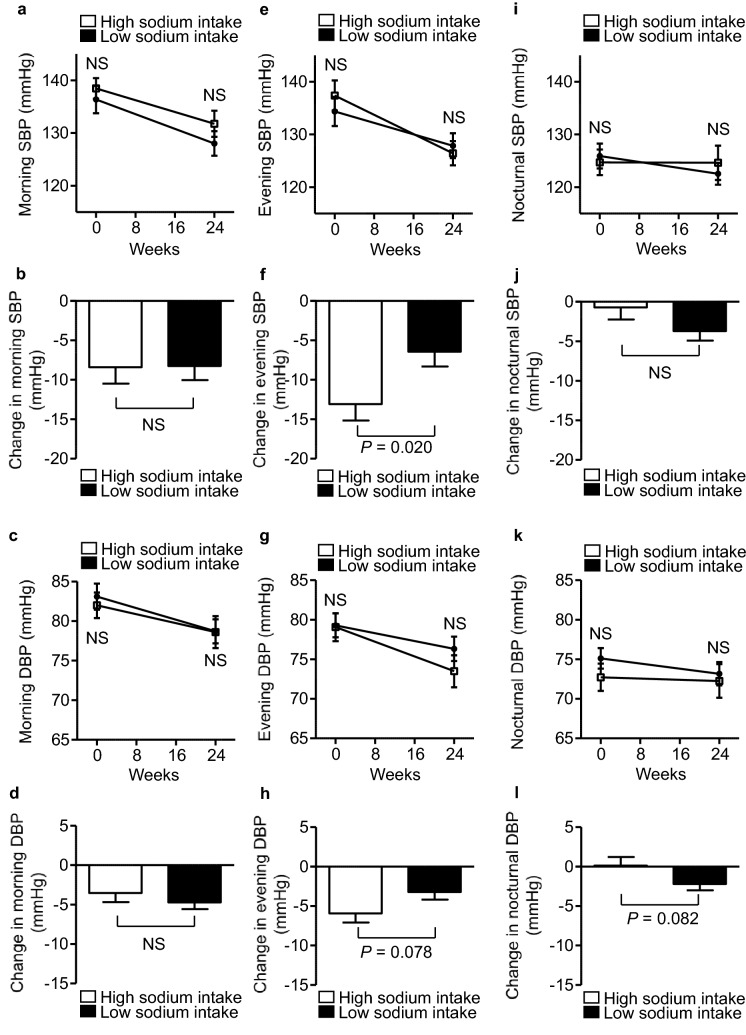
Effects of add-on dapagliflozin therapy on home-measured blood pressure in the HS and LS groups. Home blood pressure (BP) (**(a)** morning systolic BP (SBP), **(c) **morning diastolic BP (DBP), **(e)** evening SBP, **(g) **evening DBP, **(i)** nocturnal SBP, and **(k) **nocturnal DBP) in dapagliflozin-treated patients during the 24-week study period. Change in home BP (**(b) **morning SBP, **(d)** morning DBP, **(f)** evening SBP, **(h)** evening DBP, **(j) **nocturnal SBP, and **(l)** nocturnal DBP) during the 24-week study period. Eighty-four DKD patients were allocated to a high sodium intake (HS) group (open square) or a low sodium intake (LS) group (filled square), according to their median urinary sodium-to-creatinine ratio at baseline. Values are mean ± SE. The differences in the effects of dapagliflozin treatment between the two groups were analyzed using unpaired *t*-tests. NS, not significant.

At baseline, the morning, evening, and nocturnal home systolic/diastolic BPs were similar in the two groups (morning systolic BP: 138 ± 1.94 vs. 136 ± 2.60 mmHg, *P* = 0.518; morning diastolic BP: 82 ± 1.61 vs. 83 ± 1.65 mmHg, *P* = 0.635; evening systolic BP: 137 ± 2.86 vs. 134 ± 2.78 mmHg, *P* = 0.448; evening diastolic BP: 79 ± 1.77 vs. 79 ± 1.50 mmHg, *P* = 0.924; nocturnal systolic BP: 124 ± 2.43 vs. 125 ± 2.35 mmHg, *P* = 0.727; nocturnal diastolic BP: 72 ± 1.73 vs. 75 ± 1.30 mmHg, *P* = 0.268; Fig. 3a,c,e,g,i,k). After 24 weeks of dapagliflozin treatment, compared with the LS group, the reduction in evening systolic BP from baseline was significantly larger (− 13 ± 2.08 vs. − 6 ± 1.88, *P* = 0.020; Fig. 3f), and the reduction in evening diastolic BP tended to be larger (− 5 ± 1.17 vs. − 3 ± 0.99, *P* = 0.078; Fig. 3h), in the HS group. In contrast, the changes in morning and nocturnal BP from baseline were comparable at week 24 in the two groups (Fig. 3b,d,j,l).

### Effects of add-on dapagliflozin therapy on renal endpoints and home-measured blood pressure in the high salt intake and low salt intake groups based on the Tanaka formula

**Figure 4:**
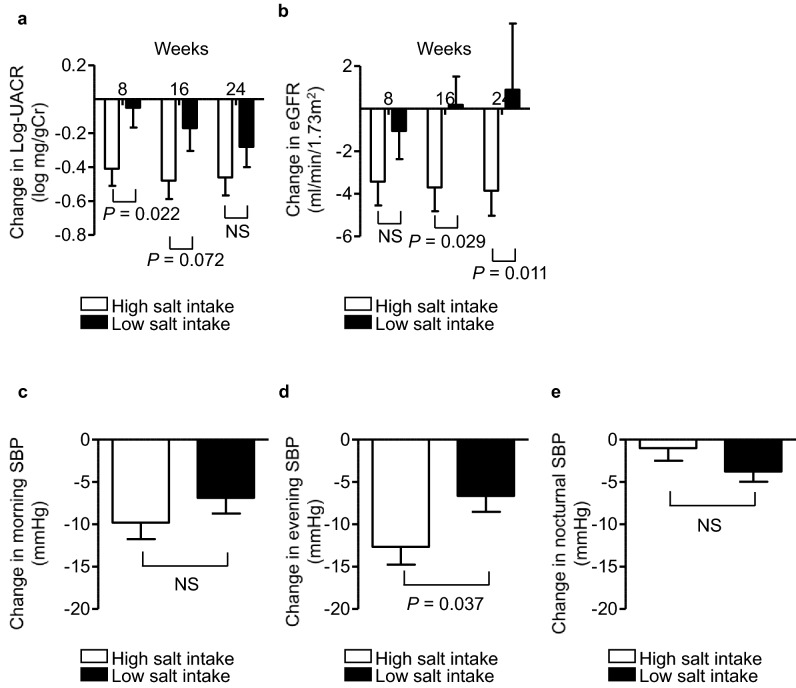
Effects of add-on dapagliflozin therapy on renal endpoints and home-measured blood pressure in the high salt intake and low salt intake groups based on the Tanaka formula. Change in **(a)** natural logarithm of urine albumin-to-creatinine ratio (ln-UACR), and **(b) **estimated glomerular filtration (eGFR) during the 24-week study period. Change in home blood pressure (BP) (**(c)** morning systolic BP (SBP), **(d)** evening SBP, and **(e)** nocturnal SBP) during the 24-week study period. Eighty-four DKD patients were allocated to the high salt intake group (open square) or low salt intake group (filled square), according to median estimated daily salt intake calculated using the Tanaka formula at baseline. Values are presented as mean ± SE. Differences in the effects of dapagliflozin treatment between the two groups were analyzed using unpaired *t*-tests at each visit. *NS* not significant.

Estimation of salt intake by spot urine was calculated using the Tanaka formula^26^. This formula can also be used to estimate salt intake in patients with CKD^27^. To assess the effects of baseline dietary salt intake on clinical variables during administration of add-on dapagliflozin, participants were divided into two groups (high and low salt intake) according to median estimated daily salt intake calculated using the Tanaka formula at baseline. Median estimated daily salt intake was 10.45 (IQR: 9.65, 12.15) and 8.06 (IQR: 7.16, 8.79) in the high and low salt intake groups, respectively (*P* < 0.001). Reductions in ln-UACR from baseline were consistent with the above results of the comparison of reductions in ln-UACR between the HS and LS groups based on urinary sodium-to-creatinine ratio at baseline (Fig. 4a). Reductions in eGFR from baseline were similar at week 8 in both groups (− 3.43 ± 1.11 vs. − 1.05 ± 1.32, *P* = 0.173), whereas reductions in eGFR at week 16 and week 24 from baseline were larger in the high salt intake group (week 16: − 3.70 ± 1.12 vs. 0.18 ± 1.33, *P* = 0.029; week 24: − 3.85 ± 1.18 vs*.* 0.89 ± 1.38, *P* = 0.011; Fig. 4b).

After 24 weeks of dapagliflozin treatment, compared with the low salt intake group, reduction in evening systolic BP from baseline was significantly larger (− 12 ± 2.10 vs. − 6 ± 1.89, *P* = 0.037; Fig. 4d) in the high salt intake group. In contrast, changes in morning and nocturnal systolic BP from baseline were comparable at week 24 in both groups (Fig. 4c,e).

## Discussion

The present study is the first to demonstrate an impact of basal sodium intake on the non-clinic measured BP-lowering effect of an SGLT2 inhibitor in albuminuric DKD patients. Four new findings were made in the present study. (i) Dapagliflozin was associated with reduction of albuminuria, regardless of basal sodium intake. (ii) The reduction in evening home systolic BP associated with dapagliflozin use was larger in the HS group than in the LS group. (iii) Dapagliflozin reduced clinic BP, and morning and nocturnal home BP, regardless of basal sodium intake. (iv) The reduction in eGFR from baseline associated with dapagliflozin use was larger in the HS group than in the LS group.

Many previous studies have shown that high sodium intake increases BP, which is a risk factor for cardiovascular and renal diseases^8^, and the restriction of sodium intake lowers BP^9–11^. High sodium intake also causes resistant hypertension^12^. Asian patients, including Japanese patients, have genetically high salt sensitivity and high dietary salt intake^28,29^. Several studies have also demonstrated that BP is salt sensitive in Japanese patients with type 2 diabetic nephropathy^13^. Furthermore, the BP-lowering effect of sodium restriction is larger in patients with diabetes mellitus (DM)^30^. Published guidelines also recommend sodium intake to be limited to 2.0 g per day (equivalent to 5.0 g salt per day) for the management of hypertension^12^. However, the mean sodium intake is higher than that recommended for sodium restriction in the general Japanese population^31^.

The use of diuretics for the treatment of hypertension is effective in patients with DM, insulin resistance, or chronic kidney disease (CKD) who have high salt sensitivity, and recommended in patients who cannot restrict their sodium intake, have excess fluid volume indicated by findings such as edema, or have resistant hypertension^32,33^. SGLT2 inhibitors are anti-hyperglycemic agents with well-characterized clinical efficacy in the treatment of T2DM. This class of drugs is also reported to exert pleiotropic effects, such as reductions in body mass, BP, and arterial stiffness, and the amelioration of aberrant endothelial function^16–20,34–37^. Furthermore, the outcomes of several recent clinical trials have indicated that SGLT2 inhibitors have cardio-renal protective effects in T2DM patients^16–20^. In the present study, we have demonstrated that the BP-lowering effect of dapagliflozin treatment is larger in patients with a high sodium intake than in those with a low sodium intake group, using BP measurements made at home.

The natriuretic effect of dapagliflozin is likely to contribute to this favorable BP-lowering effect. High dietary sodium intake promotes the expansion of extracellular fluid volume and increases cardiac output. To compensate for these hemodynamic changes and maintain BP, renal and peripheral vascular resistance falls because of an increase in the production of nitric oxide in patients with salt-resistant hypertension^38^. However, these compensatory mechanisms are impaired in patients with salt-sensitive hypertension^38^. Some previous studies have shown that the reduction in BP that is associated with dapagliflozin therapy has a natriuretic/diuretic-like effect, which is possibly associated with a reduction in plasma volume^21^. The BP-lowering effects of SGLT2 inhibitors are likely to be the result of the osmotic diuresis and mild natriuresis they induce^21,22^. The osmotic diuresis might contribute to an early reduction in BP by reducing extracellular fluid accumulation during SGLT2 inhibitor therapy^39,40^, whereas the natriuresis might play a larger role in the longer-term reduction in BP^22^.

In the present study, the reductions in UACR that were associated with dapagliflozin use were larger at weeks 8 and 16 in the HS group than in the LS group, but comparable at week 24. The UACR-lowering effect of SGLT2 inhibitors, which is partly associated with reductions in intraglomerular pressure, has been previously reported^41,42^. Similarly, RAS inhibitors reduce albuminuria in patients with diabetic nephropathy because of a reduction in intraglomerular pressure, which is induced by the drug-induced dilation of efferent arterioles^43^. However, a *post-hoc* analysis of the Reduction of Endpoints in NIDDM with the Angiotensin II Antagonist Losartan (RENAAL) and Irbesartan Diabetic Nephropathy Trial (IDNT) trials demonstrated that the UACR- and BP- lowering effects of ARB therapy are attenuated in patients with diabetic nephropathy and high sodium intake^15^. Furthermore, the greater renal and cardiovascular protective effects associated with ARB therapy, compared with non-RAS inhibitor-based therapy, were also attenuated in patients with higher sodium intake^15^. In the present study, the natriuretic/diuretic effect of dapagliflozin may, at least partially, contribute to the finding that the reduction in UACR that was associated with dapagliflozin in the HS group was comparable to that in the LS group. Recently, a pooled analysis of data from two Phase 3 trials demonstrated that tofogliflozin also reduces UACR and clinic BP in Japanese patients with T2DM regardless of their basal daily sodium intake^44^. Our findings are consistent with these results^44^. It was recently reported that 6-week treatment with dapagliflozin did not reduce proteinuria compared with placebo in 53 proteinuric nondiabetic CKD patients in a randomized, double-blind, crossover trial (DIAMOND)^45^. However, dapagliflozin caused an anticipated acute dip in iohexol-measured GFR, decreased body weight, and increased hematocrit, suggesting it caused reduced glomerular pressure and hemoconcentration with natriuresis (although BP was not significantly different between dapagliflozin and placebo treatment). It is possible the sample size was too small, and the follow-up period was too short, to detect significant changes in proteinuria in the DIAMOND study, as its design was based on studies in individuals with T2DM. All participants in the DIAMOND study had a nondiabetic kidney disease (IgA nephropathy, focal segmental glomerulosclerosis, hypertensive nephropathy, or other). Differences in underlying disease pathophysiology may have contributed to the inability to detect an antiproteinuric effect with dapagliflozin. Indeed, more recently, in the Dapagliflozin in Patients with Chronic Kidney Disease (DAPA-CKD) trial, which was a randomized, double-blind, placebo-controlled clinical trial with 4,304 CKD participants and median follow-up of 2.4 years, the risk of a renal composite outcome was significantly lower with dapagliflozin than with placebo among CKD patients regardless of the presence or absence of diabetes^46^.

In the present study, dapagliflozin had reduced eGFR to a similar extent in both groups at week 8, whereas the reduction was larger in the HS group than in the LS group at weeks 16 and 24. SGLT2 inhibitors block proximal tubular sodium reabsorption, which increases distal sodium delivery to the macula densa, thereby increasing tubulo-glomerular feedback, which leads to afferent arteriolar vasoconstriction, lower intraglomerular pressure, and less hyperfiltration in diabetic patients^47^. This is reflected in an initial transient dip in eGFR after SGLT2 inhibitor administration^16,18–20^. In the pooled analysis described above, high basal daily sodium intake positively correlated with the reduction in eGFR between the start and 52 weeks of tofogliflozin treatment^44^. The report concluded that the larger reduction in eGFR tofogliflozin-treated patients with higher sodium intake is due to the associated larger improvement in glomerular hyperfiltration^44^. In the present study, the larger reduction in eGFR in dapagliflozin-treated HS patients may be also attributed to the improvement in glomerular hyperfiltration. A difference in reduction of eGFR was not observed between the HS and LS groups at week 8 in the present study, whereas the pooled analysis showed that basal daily salt intake level was independently correlated with the change in eGFR from the 4^th^ week after tofogliflozin administration^44^. Differences in DKD stages among participants may have led to the discrepancy between our study and previous ones. In the previous pooled analysis, mean eGFR and median UACR were 83.9 ml/min/1.73 m^2^ and 16.4 mg/g Cr (IQR: 9.0, 46.0) at baseline^44^, respectively, suggesting their participants had earlier stages of DKD (where glomerular hyperfiltration may have been easier to improve) compared with participants in the present study.

Thus, the natriuresis-related hemodynamic effects of SGLT2 inhibitors play an important role in its BP-lowering and renoprotective effects. However, there are some differences between SGLT2 inhibitors and conventional diuretics. In general, diuretics such as loop diuretics and thiazides, which also have natriuretic effects, lower nocturnal BP more than daytime BP^48^. However, studies by ourselves and others have demonstrated that the reduction in BP induced by SGLT2 inhibitors persists throughout an entire day, but with a greater reduction in BP during the daytime than during the nighttime in T2DM patients^23,49,50^. In the present study, the reduction in nocturnal BP associated with dapagliflozin tended to be larger in the LS group than in the HS group, although the difference was not statistically significant. This may be partially attributed to the glucose-dependent nature of the glycemic control that is induced using SGLT2 inhibitors. The natriuretic effect of SGLT2 inhibitors may be greater during the daytime because of higher food and fluid intake. Conversely, diurnal hypertension is related to stress; therefore, an effect of SGLT2 inhibitors on sympathetic nerve activity may contribute to the greater reduction in diurnal BP^51^.

In addition to the difference in their BP-lowering effects, other diuretics, including thiazides and loop diuretics, do not have the robust cardio-renal protective effects that SGLT2 inhibitors were shown to have in the Empagliflozin Cardiovascular Outcome Event Trial in Type 2 Diabetes Mellitus Patients (EMPA-REG OUTCOME), the Canagliflozin Cardiovascular Assessment Study (CANVAS) Program, the Dapagliflozin Effect on Cardiovascular Events-Thrombolysis in Myocardial Infarction 58 (DECLARE–TIMI 58), and the Canagliflozin and Renal Events in Diabetes with Established Nephropathy Clinical Evaluation (CREDENCE) trials^16–20^. This may be because these conventional diuretics do not influence intraglomerular pressure directly^3^. Loop diuretics inhibit the Na^+^/K^+^/2Cl^−^ cotransporter in the loop of Henle, which causes an increase in the delivery of sodium to the macula densa. However, in the macula densa, the reabsorption of sodium depends on the Na^+^/K^+^/2Cl^−^ cotransporter. Therefore, loop diuretics do not affect tubulo-glomerular feedback^3^, and other diuretics, such as thiazides, amiloride, and mineralocorticoid antagonists, act too distally to affect tubulo-glomerular feedback^3^. SGLT2 inhibitors inhibit not only SGLT2 but also Na^+^/H^+^-exchanger 3 (NHE3) activity^52,53^, by which approximately 30% of filtered sodium is reabsorbed in the proximal tubule, leading to natriuresis, which triggers tubulo-glomerular feedback^3^. It has also been reported that the effects of SGLT2 inhibitors on fluid distribution may differ from those of conventional diuretics^54^. These differences in pharmacological mechanisms explain the clinical favorable outcomes of SGLT2 inhibitors.

The limitations of the present study were as follows. The most critical limitation, sodium intake, as estimated using urinary sodium excretion on the basis of single-spot urine sampling, may have contributed to large variations in the data. Some studies reported that urinary sodium-to-creatinine ratios derived from spot urine sampling correlated weakly with those derived from 24-h urine collection^55^. Multiple reports suggest sodium intake calculated using the Tanaka formula is biased with overestimation at lower levels and underestimation at higher levels^55–59^. A method such as 24-h urine collection may have provided a more accurate evaluation of sodium intake. Second, the study may have been underpowered to identify the differences in the effects of dapagliflozin according to sodium intake because the sample size calculation was based on the expected changes in ln-UACR^23^. Third, because the present study was a retrospective analysis of Y-AIDA study data, a cause and effect relationship could not be established. It is possible that sodium intake was only a confounding factor of other parameters affecting the reaction to SGLT2 inhibitors, because the HS group experienced significantly longer durations of diabetes and higher levels of HbA1c. Moreover, eGFR and BMI were higher in the HS group, although they were not significantly different at baseline. Other studies showed that SGLT2 inhibitors function more efficiently in patients with higher HbA1c levels and longer history of diabetes^60^. However, in the DECLARE-TIMI 58 trial, subgroup analysis of the renal composite outcome showed that favorable effects of dapagliflozin on renal composite outcomes in T2DM patients were consistent across baseline HbA1c levels and diabetes durations^61^. Regarding DKD patients in the CREDENCE study, reduction of risk for renal composite outcome with canagliflozin was also apparent in subgroups of patients with well-controlled diabetes, suggesting the effect of SGLT2 inhibitors on kidney function is unlikely to be mediated by further improvement of glycemic control^62^. Fourth, we cannot exclude the possibility that inclusion in the study resulted in participants changing lifestyle habits that influence nutrition, sodium intake, and glycemic control, all of which affect changes in albuminuria and BP. Fifth, extracellular fluid status was not assessed in the present study. Some previous studies have shown that the reduction in extracellular fluid volume achieved using SGLT2 inhibitors is transient^40^, and we did not assess whether dapagliflozin treatment reduced extracellular fluid volume throughout the present study period.

In conclusion, among Japanese DKD patients, there was no significant difference in reduction of UACR by dapagliflozin between the HS and LS groups. Furthermore, the BP-lowering effects of dapagliflozin, assessed using BP measurements made at home, were larger in the HS group than in the LS group, and this occurred alongside a larger reduction in eGFR, which suggests a dapagliflozin-induced improvement in glomerular relative hyperfiltration in the HS group.

## Data Availability

The datasets analyzed during the current study are available from the corresponding author on reasonable request.
